# Photography tele-transmission by regular ambulance staff for the management of mild traumatic injury: the NiCEPHORE randomised-controlled trial

**DOI:** 10.1186/s13049-022-01026-0

**Published:** 2022-10-14

**Authors:** E. Magimel-Pelonnier, N. Marjanovic, R. Couvreur, B. Drugeon, O. Mimoz, J. Guenezan

**Affiliations:** Emergency Department and Pre-Hospital Care, University Hospital of Poitiers, Service des Urgences, CHU de Poitiers, 2 rue de la Mileterie, 86000 Poitiers, France

**Keywords:** Trauma, Pre-hospital care, Tele-medicine, Emergency department

## Abstract

**Background:**

Handling emergency calls in French emergency medical call centres (EMCCs) can be challenging considering the frequent lack of relevant information. Tele-transmission device use in regular ambulances seems like a good solution to provide the EMCC physician with a more accurate assessment of the scene, particularly for mild traumatic injury (MTI). We measured the impact of ambulance staff tele-transmitted photography on prehospital dispatching optimisation for patients calling the EMCC with MTI.

**Methods:**

We conducted a prospective, single-centre, randomised-controlled trial comparing two groups of patients calling the EMCC with MTI who were or were not allocated to photography tele-transmission by ambulance staff. The primary outcome was the proportion of patients referred away from the nearest hospital (left at home for outpatient care; referred to a higher-level hospital; handled by a medical intensive care ambulance for advanced pre-hospital care) used as a marker of better orientation.

**Results:**

Between 29 April 2019 and 21 July 2020, 165 patients were randomised and 152 analysed. Photography tele-transmission resulted in better patient dispatching (24/73 [33%] patients in the intervention group vs 9/79 [11%] patients in the control group, OR 3.80 [1.63–8.90]; *p* = 0.03), without increasing the proportion of patients initially left at home for outpatient care and visiting an ED within 10 days for secondary trauma-related care (1/14 [7%] vs 1/4 [25%], OR 0.25 [0.01–24.1]; *p* = 0.41). The proportion of patients unnecessarily referred to an ED was 7% [4/59 patients] in the intervention group vs 16% [12/75 patients] in the control group (OR 0.38 [0.09–1.36]; *p* = 0.10).

**Conclusion:**

Photography tele-transmission by regular ambulance staff could improve the dispatching of patients calling French EMCCs with MTI.

*Trial registration* The study is registered with Clinicaltrials.gov (NCT04034797).

## Background

French emergency medical call centres (EMCC) are constantly facing a wide variety of medical situations, ranging from minor injuries to severe life-threatening situations. These calls can be a real challenge for the physicians in charge of medical dispatching. They need to quickly assess the severity of the situation to offer the most appropriate medical response, from simple telephone advice to the dispatching of a regular ambulance (consisting in 2 paramedics with limited training, only capable of assessing vital signs and delivering low-level of initial care like putting bandages on wounds or splints on injured limbs and basic life support. Only one of them has to be certified, following a 13 week training program, while the other one only needs a driving license) or, in more severe situations, a medical intensive care ambulance (MICA, staffed by a trained certified ambulance driver, a trained nurse and an emergency physician, in a fully-equipped ambulance capable of providing any advanced life support care and resuscitation techniques) [1–4]. In most cases, they can only rely on remote questioning of the victim or witnesses and on the report of ambulance staff when a regular ambulance is dispatched to the scene. This makes it difficult to picture the scene, particularly for limb traumatic injuries, and may lead to inappropriate referral to emergency departments (EDs) or unnecessary MICA dispatching.

In a context of increasing use of EMCCs and EDs in Europe [5–7], any information that facilitates the decision of EMCC physicians is crucial [2–4, 8]. Irrelevant referral of patients with mild traumatic injuries (MTI) to the ED unnecessarily increases their already heavy workload and generates extra costs to the healthcare system [9–12]. Conversely, underestimation of the severity or complexity of the traumatic injury by the victim himself or by the ambulance staff may lead to inappropriate outpatient care or inappropriate dispatching to a local hospital without proper technical facilities [13]. The sooner the physician can identify these situations, the earlier the patient can be sent to the relevant place, saving time to treatment and avoiding costly secondary ambulance transfers [9, 14, 15].

Telemedicine is a medical practice based on the use of communication technologies. It allows at-distance medical consultation, the completion of electronic medical records and the exchange of relevant documents such as photographies and videos. Previous studies reported its value for the management of chest pain and acute coronary syndrome [16–18], stroke [19, 20] and severe injuries [21] or to assist paramedics for complex procedures [22]. As for now, tele-transmission of photographies has only been studied in very specific situations such as remote population in mountains area or high seas [23] or during simulation [24–27]. Data assessing the impact of telemedicine for the pre-hospital management of patients with MTI is lacking.

We hypothesised that tele-transmission of photography by regular ambulance staff to EMCC physicians could improve the pre-hospital dispatching of patients with MTI. Appropriate dispatching was assessed by measuring the proportion of patients dispatched elsewhere than the nearest hospital.

## Methods

The NiCEPHORE trial is an investigator-initiated, single-centre, randomised, open-label trial, conducted from April 29, 2019 to July 21, 2020. Patients were recruited at the EMCC of the Poitiers University Hospital handling 210,000 calls per year. The “South Mediterranean IV” ethics committee approved the protocol on March 15, 2019 (approval no. 190205). Patients meeting all the inclusion criteria and none of the exclusion criteria were identified by the EMCC physician and informed of the current research. Oral consent was recorded during the conversation and added to the medical files for archiving. The study is registered with Clinicaltrials.gov (NCT04034797).

We enrolled adult patients (≥ 18 years of age), free of will (not under any guardianship or liberty privation), suffering from MTI, requiring dispatch of a regular ambulance equipped with photography tele-transmission device after calling the EMCC and consenting to participate in the study. Patients with severe trauma requiring immediate medical assistance and those with head injury, on antiplatelet therapy (APT) or anti-coagulation therapy (ACT) were excluded. Head injured patients found to be receiving APT or ACT after randomisation were excluded from the study because of the need for in-hospital assessment, regardless of the severity of associated traumatic injuries, in accordance with current guidelines [28].

Each participating regular ambulance was equipped with the same pre-hospital paramedical tele-transmission device (Nomadeec®, Exelus SAS, Bordeaux, France). Reports and photography were accessible to the EMCC physician via a dedicated, secure and encrypted website. Every qualified ambulance staff member received specific training in the use of the device. The equipped ambulances were evenly distributed throughout the region, enabling global coverage of the area.

A statistician, neither involved in the selection of patients nor in the evaluation of results, provided a computer-generated numbered list using the PROC PLAN procedure in SAS® software. Randomisation was carried out by the EMCC physician using a secure internet-based randomisation system. Patients were randomly assigned (1:1) using a minimisation method without stratification into one of the two study groups: In the intervention group, one or more injury-focused photography was sent along the standardised paramedical report and analysed by the EMCC physician. In the control group, no photography was taken and only the standard report was sent to the EMCC physician.

Data collected at inclusion were age, sex, medical history and treatment, type and description of injuries, randomisation group, nearest hospital and final decision of the EMCC physician (left at home for outpatient care/sent to nearest hospital/sent to higher level hospital/handled by MICA for advanced pre-hospital medical care). Patients were phoned back 10 days later. They were asked about the course of the initial traumatic injury and whether they had to return to an ED for any initial injury-related care.

Each medical record was reviewed by an independent blinded adjudication committee, consisting of an orthopaedic surgeon, a general practitioner and an emergency physician, to determine whether the initial decision to refer the patient to an ED was appropriate.

The primary outcome was the proportion of patients dispatched elsewhere than to the nearest hospital (left at home for outpatient care / referred to a higher-level hospital/handled by MICA for advanced pre-hospital medical care).

Secondary outcomes were the proportion of patients initially left at home for outpatient care who had to visit an ED within the 10 following days for care related to the initial traumatic injury (under-triage), the proportion of patients initially dispatched to an ED who could have underwent outpatient care after review of their medical record by the independent adjudication committee (over-triage) and the proportion of patients for whom photography was of poor quality or impossible to transmit due to network issues.

### Statistical analysis

A sample size of 516 patients (n = 258 in each group) was computed based on an estimated 10% of patients referred elsewhere than the nearest hospital in the control group and 20% in the intervention group, with one-sized alpha-risk of 5%, study power of 90%, and considering a maximum patient loss or missing data of 20%.

Continuous variables are expressed as means ± standard deviation and compared using student t-test for normally distributed variables. Categorical variables are given as number and proportion and compared using the Chi-2 test. All tests were two-tailed with no adjustment for multiple testing.

Analyses were done using R statistical package version 3.6.2 or later (The R Foundation for Statistical Computing, https://www.R-project.org/). A p-value < 0.05 was considered as significant.

## Results

**Fig. 1:**
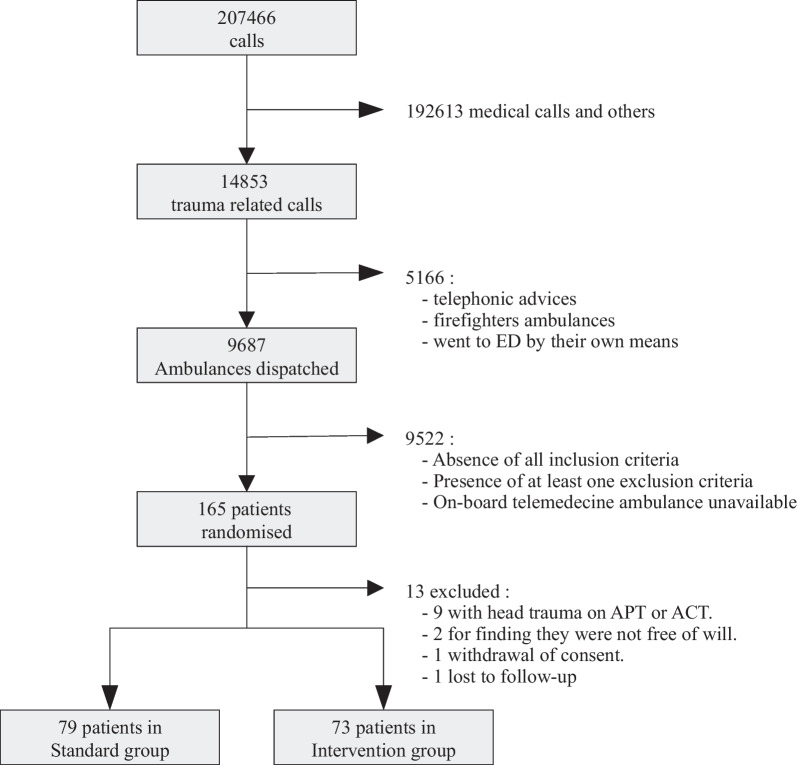
NiCEPHORE trial flow-chart. ED: Emergency Department, APT: Anti-platelet therapy, ACT: Anti-coagulant therapy

From 29 April 2019 to 21 July 2020, 207,466 emergency calls were received at the EMCC. Of these, 14,853 (7%) were trauma-related. After initial assessment, an ambulance was dispatched in 9687 (65%) cases. Due to the SARS-COV-2 pandemic, we were not able to achieve the estimated sample size. 165 (2%) patients were randomly assigned to one of the two study groups and 13 (8%) were secondarily excluded or lost to follow-up. All in all, 73 patients constituted the intervention group and 79 the control group (Fig. 1). Their demographic and clinical characteristics were broadly similar (Table 1). Photography was transmitted in all cases but was of poor quality for 2 patients (3%).

**Table 1:** : Demographic, clinical characteristics and final orientation of patients

	Standard group n = 79	Photography group n = 73	*p* value	OR [IC95%]
Age (years)	72.5 ± 22.9	67.7 ± 23	0.22	
Sex ratio M: F	0.46	0.60	0.38	
Trauma type			0.53	
Upper limb trauma	11 (14%)	8 (11%)		
Lower limb trauma	30 (38%)	21 (29%)		
Wound	34 (43%)	39 (53%)		
Others	4 (5%)	5 (7%)		
Dispatching			0.0003	
Nearest hospital	70 (87%)	49 (67%)		
Higher level hospital	2 (3%)	10 (14%)		
Ambulatory cares	4 (5%)	14 (19%)		
MICA	3 (4%)	0 (0%)		
Patients dispatched elsewhere than nearest hospital	9 (11%)	24 (33%)	0.0026	3.80 [1.63–8.90]
Over-triage	12/75 (16%)	4/59 (7%)	0.10	0.38 [0.09–1.36]
Under-triage	1/4 (25%)	1/14(7%)	0.41	0.25 [0.01–24.1]

Over-triage = proportion of patients initially dispatched to an ED that could have underwent outpatient care after patient’s medical file review

Under-triage = proportion of patients initially left at home for outpatient care that had to go to an ED within the 10 days for secondary care related to the traumatic injury

OR, Odds Ratio; MICA, Medical Intensive Care Ambulance; 95% CI, 95% Confidence Interval

All in all, 119 patients were referred to the nearest hospital, 12 to a higher-level hospital, 18 underwent outpatient care and 3 were handled by MICA. According to study group, 24 (33%) patients were referred elsewhere than to the nearest hospital in the intervention group compared with 9 (11%) in the control group (OR: 3.80 [1.63–8.90]; *p* = 0.03). After review of the medical file by the adjudication committee, the proportion of patients unnecessarily referred to an ED was 7% (4/59 patients) in the intervention group vs 16% (12/75 patients) in the control group (OR 0.38 [0.09–1.36]; *p* = 0.10). The proportion of patients initially left at home for outpatient care and visiting an ED within 10 days for secondary trauma-related care was 7% (1/14 patients) in the intervention group vs 25% (1/4 patients) in the control group (OR 0.25 [0.01–24.1]; *p* = 0.41).

## Discussion

In this randomised-controlled trial, we showed that tele-transmission of photography by regular ambulance staff was feasible and improved pre-hospital dispatching of patients calling the EMCC with MTI.

To the best of our knowledge, The NiCEPHORE trial is the first to evaluate real-time photography tele-transmission by regular ambulance as a routine care decision support for EMCC physicians. The feasibility and benefits of telemedicine in regular ambulances have previously been reported in a systematic review of the literature, especially for patients with stroke [29]. Real-time telemedicine for trauma care has been studied only in simulated situations [27], in remote areas such as high seas but without any research protocol or dedicated device [23] or as a remote guiding tool for paramedics-provided advanced care during transport of critically ill patients [24]. None of those studies were designed for routine dispatching purposes.

When any doubt subsists after questioning the patient or acknowledging the ambulance standard report, the EMCC physicians often refer the patient to the local hospital for a more complete evaluation. Conversely, we made the hypothesis that the provision of one or more injury-focused photography by the on-board telemedicine could allow the EMCC physician to better distinguish a minor traumatic injury eligible for outpatient care from a complex or more severe one requiring to be taken care of at a general hospital with appropriate technical facilities, even if it is far away. Thus, we chose the proportion of patients referred elsewhere than to the nearest hospital as the primary endpoint in the present study.

In our trial, patients allocated to photography tele-transmission were more often oriented to outpatient care. They also were more frequently referred to a higher-level hospital than those who only received a standard ambulance report. It was primarily for complex wounds and open fractures requiring specialized surgical care and, in one case, for a patient with an ocular deep wound (initially described as a periorbital wound) who was immediately dispatched to the ophthalmologist for surgical exploration whereas he was initially near a hospital without an ophthalmologist or surgical facility.

Offering outpatient care whenever possible improves patient satisfaction and decreases both the ED burden and costs to the health-care system. Similarly, referring patient to a general hospital with the most appropriate technical facilities, even if it is located further away than the local hospital, allows for faster and more accurate treatment of injuries, reducing risk of complications (such as infections…), improving functional prognosis of the injury and ultimately reducing costs for the health-care system as well [6, 7, 30, 31].

After adjudication, the use of photography tele-transmission resulted in a reduction in the proportion of patients inadequately referred to hospital, although the difference was not significant because of a lack of power in the study. The persistence of patients over-referred to the hospital despite the provision of photography could be explained by technical issues in the realisation or the transmission of photography: for 2 patients, over-triage was related to poor-quality photos that were difficult to interpret.

Conversely, only one patient (7%) in the intervention group who initially underwent outpatient care after contacting the EMCC had to visit an ED within the 10 days following the traumatic injury. This patient declined to be taken care of in an ED despite the EMCC physician’s request, but visited the ED on his own three days later.

Our study suffers from several limitations. Firstly, we had to stop inclusions after having included one third of the planned number of patients. The COVID-19 pandemic and national lockdown significantly reduced the occurrence of traumatic injuries (most of them were domestic accidents and went to the ED by their own means without calling EMCC) and greatly increased the burden on the EMCC, thereby reducing our inclusion capacity. Despite this, a significant difference in the primary outcome was observed in favour of the intervention group, due to a greater than expected advantage of the intervention. Secondly, only patients to whom an ambulance was dispatched were included. We can assume these patients were more frequently older and/or had co-morbidities. Some patients were referred to the ED not because of their traumatic injury, but because of co-morbidities or lack of autonomy. These data were taken into account by the adjudication committee. Thirdly, the generalisability of our findings is conditional on the availability of ambulances equipped with an on-board tele-transmission device and a functioning telephone network. The low inclusion rate (2%) of patients in our study can be explained by the absence of all inclusion criteria or the presence of at least one exclusion criterion in some patients. It is mainly due to work overload at the call centre and a lack of ambulances equipped with in-vehicle telemedicine available at the time of the call. Indeed, such ambulances were not dedicated for traumatic injuries but susceptible to take care of any ill patient (including COVID patients). The generalisation of the equipment to all ambulances or the use of other devices such as smartphones is expected to facilitate implementation of the technique. Finally, some randomised patients had to be secondarily excluded due to the identification of an exclusion criterion by the ambulance staff. These were mainly head injured patients receiving APT or ACT, who needed to be assessed in an ED for a head CT scan according to current recommendations [28]. We lack precise data regarding injury description insofar as our data were limited to the type and localisation of each injury. Therefore, we could not provide definitive diagnoses.

Our study also has several strengths. It was carried out in a country where dispatching after calling EMCC is carried out by physicians [1]. This allows more accurate clinical evaluation and limits the number of patients unnecessarily referred to the hospital [32, 33]. It applies a solid methodology with randomization. The comparability of the two groups is strengthened by the inclusion of patients who were exclusively managed by ambulances, all of which were equipped with the same tele-transmission system and of which the geographical distribution did not favor any subpopulation or any ED in our area.

These findings call for a medico-economic study to determine whether this approach is profitable. The cost of equipping an ambulance is not negligible (about 6000 €), but it may be compensated by the reduction of costs related to unnecessary referrals to an ED or to delayed management of complex/serious injuries when the patient is not immediately referred to the appropriate hospital.

## Conclusion

In conclusion, tele-transmission of pictures by regular ambulance staff is feasible and improves the referral of patients calling an EMCC for MTI. These promising results call for further large-scale studies not only to confirm these results in other territories, but also to demonstrate a significant reduction in the number of patients over-referred to an ED. Particularly, a study focussed on more easily accessible tools for the general population (like smartphone applications) could be very useful in a younger population, where over-triage is probably even greater.

## Data Availability

Data are available from the authors upon reasonable request and with permission of corresponding author.

## Abbreviations

ACT: Anti-coagulant therapy
APT: Anti-platelet therapy
ED: Emergency department
EMCC: Emergency medical communication center
MICA: Medical intensive care ambulance
MTI: Mild Traumatic Injury
OR: Odds-ratio
95% CI: 95% Confidence interval
